# Use of Rotorod as a Method for the Qualitative Analysis of Walking in Rat

**DOI:** 10.3791/1030

**Published:** 2008-12-10

**Authors:** Ian Q. Whishaw, Katie Li, Paul A. Whishaw, Bogdan Gorny, Gerlinde A. Metz

**Affiliations:** Department of Psychology and Neuroscience, Canadian Centre for Behavioural Neuroscience, University of Lethbridge

## Abstract

The rotorod test, in which animals walk on a rotating drum, is widely used to assess motor status in laboratory rodents. Performance is measured by the duration that an animal stays up on the drum as a function of drum speed. Here we report that the task provides a rich source of information about qualitative aspects of walking movements. Because movements are performed in a fixed location, they can readily be examined using high-speed video recording methods. The present study was undertaken to examine the potential of the rotorod to reveal qualitative changes in the walking movements of hemi-Parkinson analogue rats, produced by injection of 6-hydroxydopamine (6-OHDA) into the right nigrostriatal bundle to deplete nigrostriatal dopamine (DA). Beginning on the day following surgery and then periodically over the next two months, the rats were filmed from frontal, lateral, and posterior views as they walked on the rotorod. Behavior was analyzed by frame-by-frame replay of the video records. Rating scales of stepping behavior indicated that the hemi-Parkinson rats were chronically impaired in their posture and in the use of the limbs contralateral to the DA-depletion. The contralateral limbs not only displayed postural and movement abnormalities, they participated less in initiating and sustaining propulsion than did the ipsilateral limbs. These findings not only reveal new deficits secondary to unilateral DA-depletion, but also show that the rotorod can provide a robust tool for the qualitative analysis of movement.

**Figure:**
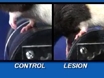


## Protocol

### Rotorod walking apparatus

The rotorod was a rotating cylinder, 4 cm in diameter, fixed 35 cm above the ground and enclosed by transparent Plexiglas. The rotorod cylinder was covered in textured rubber coating, which facilitates traction. The floor of the apparatus was covered with a layer of foam to prevent injuries when animals fell. A small electric motor provided power to turn the rotorod via a rubber belt. The rotorod was set to rotate once every 5 s.

### Video recordings and kinematics

A high-speed digital video camera was used to film the animals, using a shutter speed of a 1000 of a second.  The front, lateral, and rear views were filmed while the animal was walking on the rotorod. A two-arm Nikon, MII cold light source and one set of fiber optic lights were used to provide adequate lighting during recording. The tapes were analyzed frame-by-frame using a Sony DSR 20 DV CAM deck. Video frames were captured using a frame grabber and Macintosh computer.

### Testing procedure and analysis

In order to evaluate the differences between the walking movements of the 6-OHDA and control rats, the video records from post-surgical days 15 - 30 (after any recovery from the lesion might be expected to be largely complete) were examined by two independent observers blind to the experimental conditions. The nigrostriatal 6-OHDA lesion was induced in the right hemisphere, resulting in lasting impairments in the left fore- and hind limbs. A rating scale was developed to rate the rats’ posture and the stepping movements of the forelimbs (Whishaw et al., 2003). Five components of stepping were rated:

**posture**,  the rating of posture was made by examining the position of the rat on the drum;
**lift and release**,  the portion of the stepping cycle in which the paw was lifted from the drum;
**carry**,  the portion of the stepping cycle in which the limb was held above the drum as the rat moved forward;
**advance**,  the portion of the reach in which the limb was extended to regain contact with the drum; and
**placement**,  in which the paw was placed back on the drum.

The following criteria were used to define a normal movement:

** Posture:** The body of the rat was oriented over the drum with the head held up.
**Lift and release:** The shoulder, elbow and wrist are flexed raising the paw from the surface of the drum.
**Carry:** The paw is held above the surface of the drum, the digits are loosely flexed, and the digit tips are centered on the midline of the body.  Advance: The limb is extended forward with the digits moving from a semiflexed position to an extended and open position.
**Placement:** The limb is extended at the shoulder, elbow, and wrist and the digits are opened to place the paw forward and on the surface of the drum, with digit 2 (second digit from the midline) located at approximately the center of the rat’s body.

Each movement was rated on a 3-point scale:  1=/the movement resembled that of a control rat, 0.5=/the movement appeared slightly abnormal, and 0=/the movement was clearly abnormal (Whishaw et al., 2003). This rating scale was then used to evaluate five stepping movements from each rat on postoperative days 1, 3, 5, 7, 9, and 15. The scores from the five measures were averaged for each rat on each day, and the results subjected to an ANOVA with days as a repeated measure.

### Overview of rotorod walking in 6-OHDA and control rats

The movement rating scale was applied to the postoperative test sessions for qualitative movement analysis. During the test sessions, the 6-OHDA lesion group received significantly lower scores than did the control group, F(1,8)=33.2, p<0.001, but there was no significant effect of Test Day, nor was there a significant  Group by Test Day interaction. The scores of stepping were then separately analyzed with the forelimb as a variable (for the 6-OHDA rats the forelimb contralateral to the lesion versus the forelimb ipsilateral to the lesion; for the control rats, the left paw vs. the right paw). There was a significant effect of Forelimb, F(1,40)=15.8, p<0.01, and there was a significant interaction of Group by Forelimb, F(1,40)=15.0, p<0.01. Follow-up t-tests indicated that the contralateral-to-lesion forelimb of the 6-OHDA group received significantly lower scores than the ipsilateral-to-lesion forelimb or the forelimbs of the control rats, which did not differ. In order to determine which components of the movement contributed to the poor scores obtained by the 6-OHDA group, the measures of posture and the individual components of the stepping cycle from the ipsilateral and contralateral forelimbs of the 6-OHDA group were compared to the values obtained from the control rats. The 6-OHDA group differed in posture and all of the stepping components made by the contralateral limb, but none of the components of the ipsilateral limb (p<0.05).

 

### Qualitative and descriptive differences in posture in control and 6-OHDA rats

In a control rat, the head is held in a horizontal position, and the supporting forelimb and hind limbs straddle the drum forming a supporting arch. The 6-OHDA rats typically walked with the head down with their impaired forelimbs (facing the viewer) positioned closer together and higher up on the drum surface than occurred for the ipsilateral limbs or the limbs of control rats (see video for a control rat with score "1" for posture, and a 6-OHDA lesion animal with a score of "0"). Typically, the abnormality in posture can be seen at a glance from the relative position of a rat’s head.

### Forelimb stepping

In the control sequence, the stepping forelimb is lifted by flexing the shoulder, elbow and wrist. As the limb is lifted, the digits are loosely flexed, and the limb is lifted well above the surface of the drum. When the limb is advanced, it is extended at the shoulders, elbow and wrist and as it is placed the digits are extended and open. In comparison to the control rats, the amplitude of all components of the DA-depleted rats contralateral to lesion limb is attenuated. The limb releases the drum later, is lifted less, and is advanced a shorter distance than is the control forelimb. In addition, when the limb is lifted, the digits are flexed more, and when the limb is placed, the digits are extended and opened less than those of a control rat. The control forelimb is lifted once it reaches the top of the drum, the wrist is flexed to lift the paw up and forward, and the wrist is extended as the limb advances. By contrast, the 6-OHDA rat’s contralateral to lesion forelimb is carried further caudally by the movement of the drum, and thus releases later with the wrist still extended. Flexion of the wrist appears to be passive and occurs as the digits are carried backward by the movement of the drum, and when the forelimb is brought forward, the paw and forearm are not lifted clearly off the drum surface. The forelimb of a control rat extends well forward and the digits open as the limb advances. As the limb is placed, it is placed with an arpeggio movement, with digit 5 contacting the surface of the drum first, followed successively by digits 4, 3, and 2. By contrast, the limb of the 6-OHDA rat is lifted and extended less, the digits are extended less, and they contact the surface of the drum almost concurrently with a ‘slap’. The video shows forelimb stepping movements in a control animal that were scored as "1", and forelimb stepping movements in a 6-OHDA lesion animal that were mainly scored as "0".

### Hind limb stepping

There were also impairments in the contralateral-to-lesion hind limbs of the 6-OHDA rats. As the control hind limb moves backward, it appears to push against the turning drum and as it does so, the toes rotate outward. As the limb moves forward, the foot is swung outward. The hind limb of the 6-OHDA rat moves directly backward with drum rotation and if anything, turns slightly inward. As the limb moves forward, the foot is carried directly forward. The foot of the control rat swings outward and as it is advanced, the toes are held higher than the ankle, and the toes extend. During the control rat’s placement, toe 5 contacts the surface of the drum first and weight is distributed across the toes and palm.  In contrast, the toes of the 6-OHDA rat are flexed during carry and placement is made without an arpeggio movement. The toes of the control rat are released in a reversed arpeggio pattern as toe 5 is lifted first and toe 1 is lifted last. This movement was missing in the 6-OHDA lesion rat while the ankle maintained its flexed posture during the carry movement. Again the advance movements of the 6-OHDA rat are attenuated, with the limb less exorotated, lifted less, and advanced less than that of the control rat. As control rats place their toes, they are extended and opened, such that toe 5 points laterally relative to the body of the rat. Toe 5 contacts the drum first followed by the remaining toes such that the rat supported its weight on the toe tips. For the 6-OHDA rat, the toes are somewhat flexed as the foot is carried forward, the toes are lower to horizontal relative to the ankle, there is less opening of the toes when the foot is placed on the drum. Stride length is also somewhat shorter than that of the control rats. In order to quantify the differences between control and 6-OHDA hind limb movements, the components of lift and release, carry, advancement, and placement were rated on a 3-point scale, as were the forelimbs. The measures were made from video records made on post surgical day 20, and three step cycles were rated for each rat. There were significant Group differences, F(1,8)=47.6, p<0.001. Follow-up t-tests on the components indicated that all components of the control rats received significantly higher scores than did the components of the 6-OHDA rats. The video shows hind limb stepping movements in a control animal that were scored as "1". Hind limb stepping movements in the 6-OHDA shown in the video were mainly rated with a score of "0".

## Discussion

The purpose of the present study was to examine whether the rotorod test for rodent locomotion skill can be used for the qualitative examination of posture, forelimb stepping and hind limb stepping in nervous system injured rats. For the analysis, rats with unilateral DA depletions and control rats were video recorded from front, lateral and posterior views. A rating scale of posture and forelimb movements indicated that stepping movements were chronically impaired following surgery. Examination of limb movements indicated that whereas the DA-depleted rats could use the limbs contralateral to the lesion for support, they received minimal use for shifting weight. The results of this study indicate that the rotorod task, in addition to providing quantitative measures of motor impairments, can also provide insights into the qualitative impairments.

The rotorod task has been widely used for assessing the motor status of rodents.  To date the major measure taken from the task are end point measures, measures of the speed/duration that an animal is able to walk. We felt that there are attributes of the task that might make it ideal for the qualitative examination of walking movements. First, because an animal must balance and step, the task involves considerable skill and may thus reveal impairments that may not be apparent in simpler tasks of overground locomotion (Schallert et al., 1992; Olsson et al., 1995; Chang et al., 1999; Muir and Whishaw1999b; Metz et al., 2005). The sensitivity of this task and the video analysis method is demonstrated by the presence of impairments in acute and chronic stages after the lesion. Second, animals need not be food or water deprived to motivate behavior, as required for the study of skilled forelimb reaching (Miklyaeva et al., 1994). Third, the task requires minimal training. For example, we were able to film stepping movements as soon as the animal was placed in the apparatus. Fourth, because the animal remains in place while stepping, it is easier to video record movements from a variety of angles. Fifth, the analysis method allows evaluation of qualitative aspects of walking movements with simple rating systems. The rating systems produce reliable results even in one recording sequence from an animal and thus are time-efficient for the experimenter.

By developing a simple 3-point rating scale of posture, and for four components of the stepping cycle (lift, carry, advance, and place) it was possible to identify impairments in the contralateral-to-lesion limbs of the DA-depleted rats as early as one day following surgery. The impairment appeared chronic because the impairment was similar at 15 days after surgery, and in informal tests given a number of months after surgery. Although our measures were derived from as many as five stepping cycles per rat, the impairment was sufficiently robust to be identified from a single stepping cycle in either the forelimbs or hind limbs. The impairment in use of the limbs was also apparent in the posture of the animals so that a simple inspection of the posture of the animal could reveal that it had an impairment.

A number of previous studies have identified the source of the impairment in the limbs of 6-OHDA lesion animals (Miklyaeva et al., 1995; Morrissey et al., 1989; Muir and Whishaw, 1999a; Schallert et al., 1979). The impaired limbs are able to support body weight and the allied reflexes required to maintain posture are present. Thus animals are able to stand on the limbs, and they brace against attempts to displace them. The limbs are used to regain postural support, when support is lost. Thus animals will step or hop to regain support when they lose their balance. During walking or turning, the limbs participate in stepping, but only when the momentum of the animal displaces the limb from its role in postural support. Thus, during overground locomotion the unilateral lesion animal ‘limps’ as its impaired limbs catch-up as the animal’s forward movement displaces the limb from postural support. When turning, the animal pivots on its impaired hind limb when turning ipsilateral to the impaired limb, and it ‘falls’ when turning contralateral to the impaired hind limb to displace it from its supporting action. We were able to observe very similar impairments, weight support but not weight shifting, in the contralateral- to-lesion limbs of the rats that received unilateral 6-OHDA lesions as they walked on the drum. Although both the forelimb and hind limb were used for postural support, neither limb appeared to be used for weight shifting. For example, both the forelimb and hind limb were lifted from the drum much later than occurred for the unimpaired limb, or the limbs of control rats. When the movement of the drum displaced the limbs from their supporting functions, their forward movement was attenuated, moving only the necessary distance to support body weight. Neither the impaired forelimb nor the hind limb appeared to lift the body sufficiently so that its pair mate could swing clear of the drum surface. The limbs also did not appear to participate in shifting the rats’ weight forward. For example, whereas the hind limb of control rats abducted and pushed against the drum to propel the animal forward, the rats did make similar movements with their impaired hind limb.

We were also able to observe some novel features of rat stepping movements and thus novel impairments in the way in which the DA-depleted rats moved their impaired limb. Both the forelimb and the hind limb contacted the drum surface with an arpeggio movement in which the outside digit (digit 5) contacted the drum surface first, followed successively by digits 4, 3, and 2. The impaired forelimb and hind limb did not appear to similarly locate and anticipate the drum surface, and so contacted the surface with a flatter palm-down movement.  A similar arpeggio movement has been described in the forepaw of rats as they place their forepaw to grasp food (Whishaw and Gorny, 1994), and this movement is also impaired in the impaired limb of DA-depleted rats (Miklyaeva et al., 1994). In summary, the results of the present study indicate that that the rotorod can provide a very useful way of examining posture and stepping in brain injured rats. The task demands balancing and skilled stepping movements, requires minimal training, no motivating food reward, and is easy to score and film. Additionally, impairments observed on the rotorod can be generalized to other test situations.
